# *Arcanobacterium pyogenes* Sepsis in Farmer, Brazil

**DOI:** 10.3201/eid1507.081072

**Published:** 2009-07

**Authors:** Carlos Emilio Levy, Rogerio Jesus Pedro, Angela Von Nowakonski, Luciana Maria Holanda, Marcelo Brocchi, Marcelo Carvalho Ramos

**Affiliations:** Universidade Estadual de Campinas, Campinas, São Paulo, Brazil

**Keywords:** Arcanobacterium pyogenes, sepsis, otitis media, bacteria, letter

**To the Editor: ***Arcanobacterium pyogenes* is a normal inhabitant of the mucous membranes of domestic animals, such as cattle, sheep, swine, and goats (*1*). Diseases caused by this agent have been reported for persons who live in rural areas and have underlying illnesses such as cancer and diabetes (*2*–*4*). A recent literature review (*3*), elicited by a case of *A. pyogenes* endocarditis, found 13 unequivocal cases of human infection with this agent; many patients had a history of close contact with domestic animals. However, septicemia was not reported.

In June 2006, a 27-year-old immunocompetent man was hospitalized in Campinas (São Paulo, Brazil) for fever, cough with purulent bloody sputum, and discharge from and pain in both ears. The patient was a farmer who lived in the rural Amazon area and had extensive contact with cattle and swine. For the past 3 days he had been taking amoxicillin, 1.5 g/day, for chronic otitis media. At the time of hospital admission, his temperature was 38.9°C, respiratory rate 24 breaths/min, and blood pressure 100/70 mm Hg. He had palpable hepatosplenomegaly, but no murmur was detected in the precordium. Computed tomography (CT) scan of his chest showed multiple pulmonary nodules and alveolar infiltrates with small cavities suggestive of septic infarctions. Abdominal CT scan confirmed hepatosplenomegaly with focal lesions in the spleen. CT scan of the middle ear showed bilateral cholesteatoma and mastoiditis. No abnormalities were found during 2 transthoracic echocardiography procedures. Laboratory values were PaO_2_ 63.4 mm Hg, hemoglobin 11.9 g/dL, thrombocytes 109 × 10^3^/mm^3^, leukocytes 11.05 × 10^3^/mm^3^ with a left shift, serum albumin 2.6 g/dL, alanine aminotransferase 108 U/L, and total bilirubin 2.57 mg/dL. Blood and the ear secretion were submitted for culture. Cefepime was prescribed, 2 g/twice a day.

Three blood cultures grew gram-positive bacilli, initially identified as *Corynebacterium* spp., sensitive to penicillin, ampicillin, ceftriaxone, gentamicin, clindamycin, vancomycin, and resistant to erythromycin, as determined by disk-diffusion test. Ear discharge culture grew *Proteus mirabilis,* sensitive to β-lactams, cephalosporins, and aminoglycosides. Subcultures of the gram-positive bacilli on sheep blood agar grew pinpointed, grayish, β-hemolytic colonies, identified as *A. pyogenes* by use of API Coryne 2.0 kit (bioMérieux, Durham, NC, USA; code 4732761). Although susceptibility standards are not available for this organism, it was considered susceptible to penicillin and ampicillin by combining the disk-diffusion test with the MICs, as determined by the Etest (0.06 mg/L for penicillin, 0.023 mg/L for ampicillin).

Partial 16S rDNA was amplified by using primers p27f and BAC1401r and sequenced by using primers 1100r, 765fs, and 10f. Sequences were compared with those available in GenBank by using gapped BLASTN 2.0.5 software (*5*). Identification to the species level was defined as a 16S rDNA sequence identity >97%. Phylogenetic analysis was performed by using MEGA version 4.0 (*6*) after multiple alignments of data by ClustalX (*7*); gaps were treated as missing data. Clustering was performed by the neighbor-joining method (*8*). Bootstrap analysis was used to evaluate tree topology of the neighbor-joining data by performing 1,000 resamplings (*9*). BLASTN analysis of the 16S sequence of the isolate showed 99% identity with the 16S sequence of *A. pyogenes* (accession no. X79225). Phylogenetic analyses with MEGA grouped this isolate with *A. pyogenes* NCTC 5224 in a branch separated from other species; this grouping supported the phenotypic identification.

During the 7 days after admission, the patient’s condition worsened, cefepime was withdrawn, and ampicillin 6 g/day plus gentamicin 240 mg/day were prescribed. The patient became afebrile, gradually recovered, and was discharged after 28 days of therapy.

Our patient had otitis media that progressed to sepsis, which was diagnosed by clinical, laboratory, and imaging findings. The causative agent may have been undetectable in ear discharge if it was overshadowed by a strain of *P. mirabilis,* a fastidious organism that also colonizes or co-infects this site. Endocarditis could not be ruled out because transesophageal echocardiography was not available.

*A. pyogenes* is usually susceptible to benzyl penicillin, ampicillin, gentamicin, and macrolides and resistant to trimethoprim/sulfamethoxazole, streptomycin, and tetracyclines (*10*). The isolate from this patient was sensitive to β-lactams, ceftriaxone, and gentamicin. However, susceptibility standards are not available because *A. pyogenes* rarely causes disease in humans. The patient had taken oral amoxicillin before admission, but his condition had not improved; subsequent addition of cefepime was also unsuccessful. The organism was probably sensitive to ampicillin, considering the low MIC and the expected serum concentration of the drug, but diffusion into the middle ear may have been poor or the local conditions caused by the cholesteatoma may have influenced the poor outcome of initial therapy. Treatment with intravenous ampicillin plus gentamicin produced full recovery.

Clinical laboratories do not routinely attempt to identify this organism. However, even in the absence of substantial concurrent illness, *A. pyogenes* must be considered as an etiologic agent of several human infections, especially septicemia, for patients with a history of close contact with domestic animals, mainly cattle and swine.
